# Custodiol Versus Del Nido Cardioplegia in Minimally Invasive Mitral Valve Repair–a Propensity Score-Matched Study

**DOI:** 10.1093/icvts/ivag074

**Published:** 2026-03-04

**Authors:** Dustin Greve, Serdar Akansel, Jan Holler, Martina Dini, Emilija Miskinyte, Hristian Hinkov, Stephan Jacobs, Volkmar Falk, Jörg Kempfert, Markus Kofler

**Affiliations:** Department of Cardiothoracic and Vascular Surgery, Deutsches Herzzentrum der Charité, 13353 Berlin, Germany; Charité – Universitätsmedizin Berlin, corporate member of Freie Universität Berlin and Humboldt-Universität zu Berlin, 10117 Berlin, Germany; Department of Cardiothoracic and Vascular Surgery, Deutsches Herzzentrum der Charité, 13353 Berlin, Germany; Charité – Universitätsmedizin Berlin, corporate member of Freie Universität Berlin and Humboldt-Universität zu Berlin, 10117 Berlin, Germany; Department of Cardiothoracic and Vascular Surgery, Deutsches Herzzentrum der Charité, 13353 Berlin, Germany; Charité – Universitätsmedizin Berlin, corporate member of Freie Universität Berlin and Humboldt-Universität zu Berlin, 10117 Berlin, Germany; Department of Cardiothoracic and Vascular Surgery, Deutsches Herzzentrum der Charité, 13353 Berlin, Germany; Charité – Universitätsmedizin Berlin, corporate member of Freie Universität Berlin and Humboldt-Universität zu Berlin, 10117 Berlin, Germany; Department of Cardiothoracic and Vascular Surgery, Deutsches Herzzentrum der Charité, 13353 Berlin, Germany; Charité – Universitätsmedizin Berlin, corporate member of Freie Universität Berlin and Humboldt-Universität zu Berlin, 10117 Berlin, Germany; Department of Cardiothoracic and Vascular Surgery, Deutsches Herzzentrum der Charité, 13353 Berlin, Germany; Charité – Universitätsmedizin Berlin, corporate member of Freie Universität Berlin and Humboldt-Universität zu Berlin, 10117 Berlin, Germany; DZHK (German Centre for Cardiovascular Research), 10785 Berlin, Germany; Structural Heart Interventions Program (SHIP), Deutsches Herzzentrum der Charité (DHZC), 13353 Berlin, Germany; Department of Cardiothoracic and Vascular Surgery, Deutsches Herzzentrum der Charité, 13353 Berlin, Germany; Charité – Universitätsmedizin Berlin, corporate member of Freie Universität Berlin and Humboldt-Universität zu Berlin, 10117 Berlin, Germany; Department of Cardiothoracic and Vascular Surgery, Deutsches Herzzentrum der Charité, 13353 Berlin, Germany; Charité – Universitätsmedizin Berlin, corporate member of Freie Universität Berlin and Humboldt-Universität zu Berlin, 10117 Berlin, Germany; DZHK (German Centre for Cardiovascular Research), 10785 Berlin, Germany; Department of Health Science and Technology, Swiss Federal Institute of Technology, 8092 Zurich, Switzerland; Department of Cardiothoracic and Vascular Surgery, Deutsches Herzzentrum der Charité, 13353 Berlin, Germany; Charité – Universitätsmedizin Berlin, corporate member of Freie Universität Berlin and Humboldt-Universität zu Berlin, 10117 Berlin, Germany; Department of Cardiothoracic and Vascular Surgery, Deutsches Herzzentrum der Charité, 13353 Berlin, Germany; Charité – Universitätsmedizin Berlin, corporate member of Freie Universität Berlin and Humboldt-Universität zu Berlin, 10117 Berlin, Germany

**Keywords:** cardioplegia, Del Nido, Custodiol, histidine-tryptophan-ketoglutarate (HTK), mitral valve repair, minimally invasive mitral valve surgery

## Abstract

**Objectives:**

Minimally invasive mitral valve repair (MIMVR) is the preferred approach to treat mitral regurgitation in specialized centres. Custodiol and Del Nido cardioplegia are widely used, yet direct comparative data on their efficacy and safety in this setting remain limited. This study investigated their efficacy and impact on early outcomes in a propensity-matched cohort.

**Methods:**

We performed a single-centre, retrospective matched cohort study of 2490 patients undergoing minimally invasive mitral valve surgery between October 2014 and January 2025. After exclusions, 960 patients entered 1:1 propensity score matching based on risk factors, baseline characteristics and procedural parameters, yielding 778 matched cases treated with Custodiol or Del Nido cardioplegia. Perioperative dynamics of cardiac enzymes were evaluated as the primary outcome measure, along with an exploratory analysis on clinical outcomes.

**Results:**

Del Nido cardioplegia was associated with lower postoperative creatine kinase and creatine kinase‑MB levels, most pronounced in the first 24 hours (*P* < .001). Del Nido yielded a lower inotrope score at 6 hours, higher perioperative sodium and fewer cardioversions for ventricular arrhythmia after declamping with fewer shocks required (all *P* < .001). Rates of major complications were similar between groups, and 30‑day mortality was 0% in both groups.

**Conclusions:**

Del Nido cardioplegia provided superior myocardial protection based on biomarker dynamics and a lower incidence of post-cross-clamp ventricular arrhythmia, while overall clinical outcomes remained comparable. It may therefore be considered the preferred single-dose cardioplegic solution for MIMVR.

## Graphical Abstract

**Figure:**
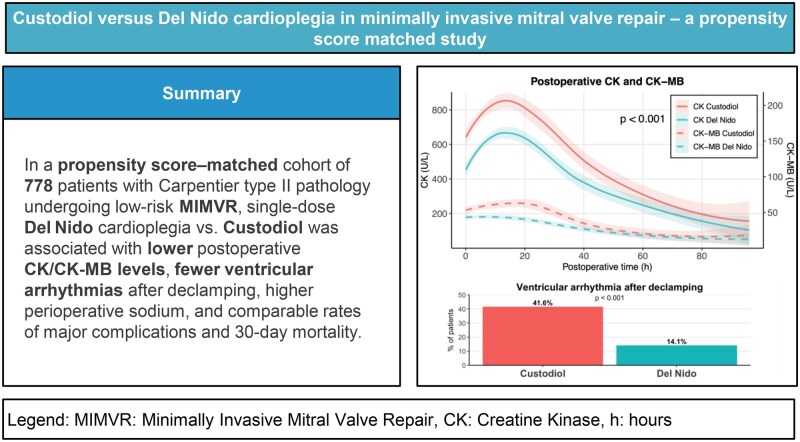

## INTRODUCTION

Mitral regurgitation is the second most prevalent valvular heart disease in developed countries and a major indication for surgery. Mitral valve repair or replacement is the definitive treatment when symptoms or ventricular dysfunction develop, with repair being the preferred option when feasible.^1^ Over the last 2 decades, minimally invasive mitral valve repair (MIMVR), usually performed via a right-sided mini-thoracotomy, has emerged as the gold standard approach in experienced centers.^2^^,^^3^ By reducing surgical trauma, MIMVR offers clinical outcomes comparable to conventional sternotomy, with shorter recovery times and reduced mortality.^4–7^

Adequate myocardial protection is critical in MIMVR, where surgical access is limited. Single-dose cardioplegic strategies are favoured to enhance surgical visualization and reduce procedural complexity.^8^ Despite the growing experience in MIMVR, optimal myocardial protection strategies have yet to be fully established.

Custodiol (Dr Franz Köhler Chemie GmbH) is an intracellular, crystalloid-based cardioplegia that induces hyperpolarizing arrest. Its key advantage lies in its capacity to provide up to 3 hours of myocardial protection with a single dose, making it well-suited for complex or prolonged procedures.^9^ Concerns persist regarding reperfusion ventricular fibrillation and dilutional hyponatraemia, and clinical evidence has not consistently demonstrated superiority over other cardioplegic solutions.^9^

Del Nido cardioplegia, a 4:1 blood-based solution initially developed for paediatric use, has increasingly been adopted in adult cardiac surgery. Del Nido is effective for 60-90 min after a single dose and requires lower total cardioplegia volumes compared to traditional strategies.^10^^,^^11^ Nonetheless, questions remain regarding its efficacy in patients with coronary artery disease and during extended ischaemic periods.

Comparative evidence in the context of minimally invasive mitral valve surgery (MIMVS) remains limited but growing. A recent propensity-matched study of MIMVS patients (*n* = 312) found modified Del Nido and Custodiol to offer equivalent myocardial protection with similar rates of ischaemic events.^12^ For all cardiac procedures, a recent network meta-analysis across various cardioplegia types suggested a possible lower perioperative mortality for adults undergoing cardiac surgery using Del Nido.^13^ Despite increasing adoption of both solutions, there is no consensus on which single-dose cardioplegia provides superior myocardial protection in MIMVS.

This study aimed to directly compare Custodiol and Del Nido cardioplegia with respect to myocardial injury markers and early clinical outcomes in patients undergoing MIMVR.

## METHODS

### Study design and patient selection

**Figure 1.**
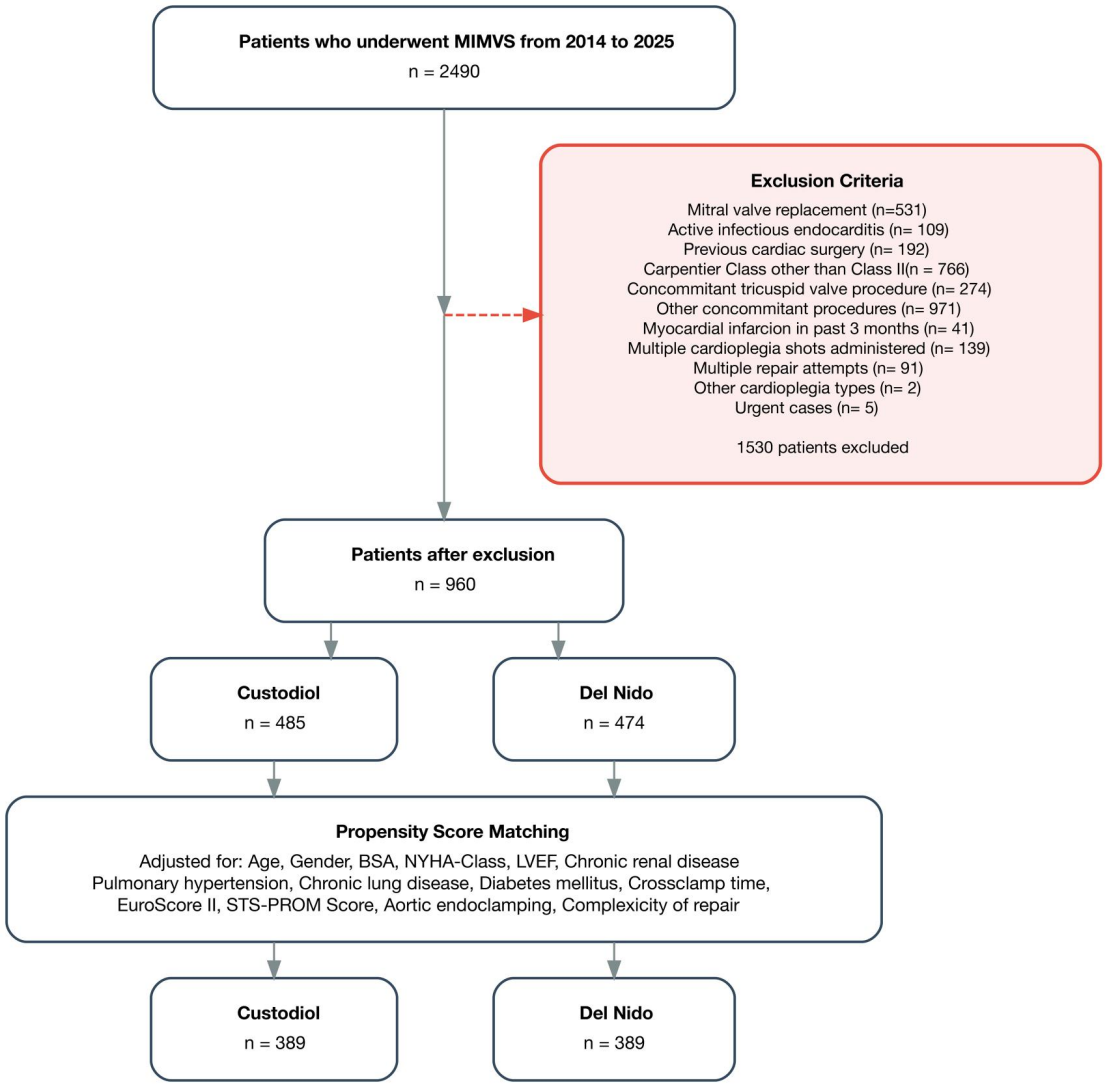
Flowchart of the Study Cohort. Individual Patients Could Meet More than One Exclusion Criterion, Resulting in Fewer Excluded Cases than Exclusion Events.

This retrospective, single-centre observational study included all patients who underwent MIMVS at the authors institution between October 2014 and January 2025. Of 2490 consecutive cases, 1530 were excluded according to predefined criteria to obtain a homogeneous cohort suitable for biomarker-based analysis. The exclusion criteria were deliberately chosen to minimize confounding factors known to substantially influence postoperative creatine kinase (CK) and CK-MB release. Exclusion criteria were mitral valve replacement, active infective endocarditis, previous cardiac surgery, Carpentier class pathology other than type II, concomitant tricuspid or other cardiac procedures (including ablation or rhythm surgery), recent myocardial infarction (within 3 months), multiple cardioplegia doses administered, multiple repair attempts, use of cardioplegia solutions other than Custodiol or Del Nido, and non-elective (defined as planned urgent) surgeries. These criteria ensured a low-risk cohort undergoing isolated mitral valve repair under standardized single-dose cardioplegia conditions. To minimize bias and account for baseline imbalances, propensity score matching was performed. A detailed patient flow diagram is shown in Figure 1.

The study was approved by the institutional ethics committee (Approval No. EA4/041/21); informed consent was waived due to the retrospective design. In accordance with the approved ethics protocol, anonymized clinical data are stored for an indefinite duration and in compliance with the WMA Declaration of Taipei.

### Surgical technique and cardioplegia protocol

Surgical access was achieved via a 4 cm right lateral mini-thoracotomy, as previously described in detail.^14^^,^^15^ Surgeries were performed either under direct vision or fully 3D endoscopic. Patients in both groups were treated by the same pool of surgeons using standardized MVR techniques, including artificial chordae implantation, leaflet resection, and ring annuloplasty. Cardiopulmonary bypass was established via femoral cannulation, open or percutaneously, depending on vascular anatomy and surgeons’ discretion. Aortic occlusion was achieved using either a transthoracic Chitwood clamp or an intra-aortic occlusion device (IntraClude, Edwards Lifesciences Corporation) inserted via a separate femoral arterial sheath, following the technique previously reported by our institution.^16–18^ Cardioplegia was administered antegrade as a single dose through the aortic root using a dedicated catheter or via the IntraClude device.

Cardioplegic solution selection was based on institutional standards, which transitioned from Custodiol to Del Nido during the study period, with a transition phase in 2020-2021 during which both solutions were used in parallel, followed by exclusive use of Del Nido from 2022 onwards. Custodiol was administered as a single dose of 1850 mL at 4 °C, according to the manufacturer’s protocol. Del Nido was prepared by mixing its crystalloid component in a 4:1 ratio with autologous blood and delivered as a single dose of 1250 mL, administered as cold cardioplegia, with the crystalloid component stored at approximately 4 °C and mild warming occurring during preparation and blood mixing prior to delivery.

### Data collection and outcomes

Clinical and perioperative data were extracted from the institutional electronic medical record system. The primary outcome was the quality of myocardial protection, assessed by serial measurements of CK and creatine kinase-MB (CK-MB) following surgery. Blood samples were drawn according to institutional standard operating procedures (usually immediately postoperatively, at 4, 12, and 24 hours and daily until discharge, with timing varying depending on clinical workflow). The secondary outcome was the occurrence of ventricular arrhythmias after aortic declamping, operationalized as the need for intraoperative electrical cardioversion. Exploratory endpoints included postoperative complications, key features of the postoperative course, and 30-day mortality. The custom postoperative inotrope score was defined as dobutamine + dopamine + 100 × epinephrine (μg/kg/min). The rate of perioperative myocardial infarction was defined according to the criteria of the Fourth Universal Definition of Myocardial Infarction (type 5 MI).^19^

### Statistical analysis

Analyses were performed in R 4.4.1 (R Foundation for Statistical Computing) using RStudio 2024.12.1 and the tidyverse ecosystem. Because patients were matched into pairs (Custodiol vs Del Nido), all between-group comparisons were conducted as dependent (paired) tests with a significance level of α = .05. Binary outcomes were compared using McNemar’s test. Continuous outcomes were compared using the paired Student’s *t*-test.

**Figure 2.**
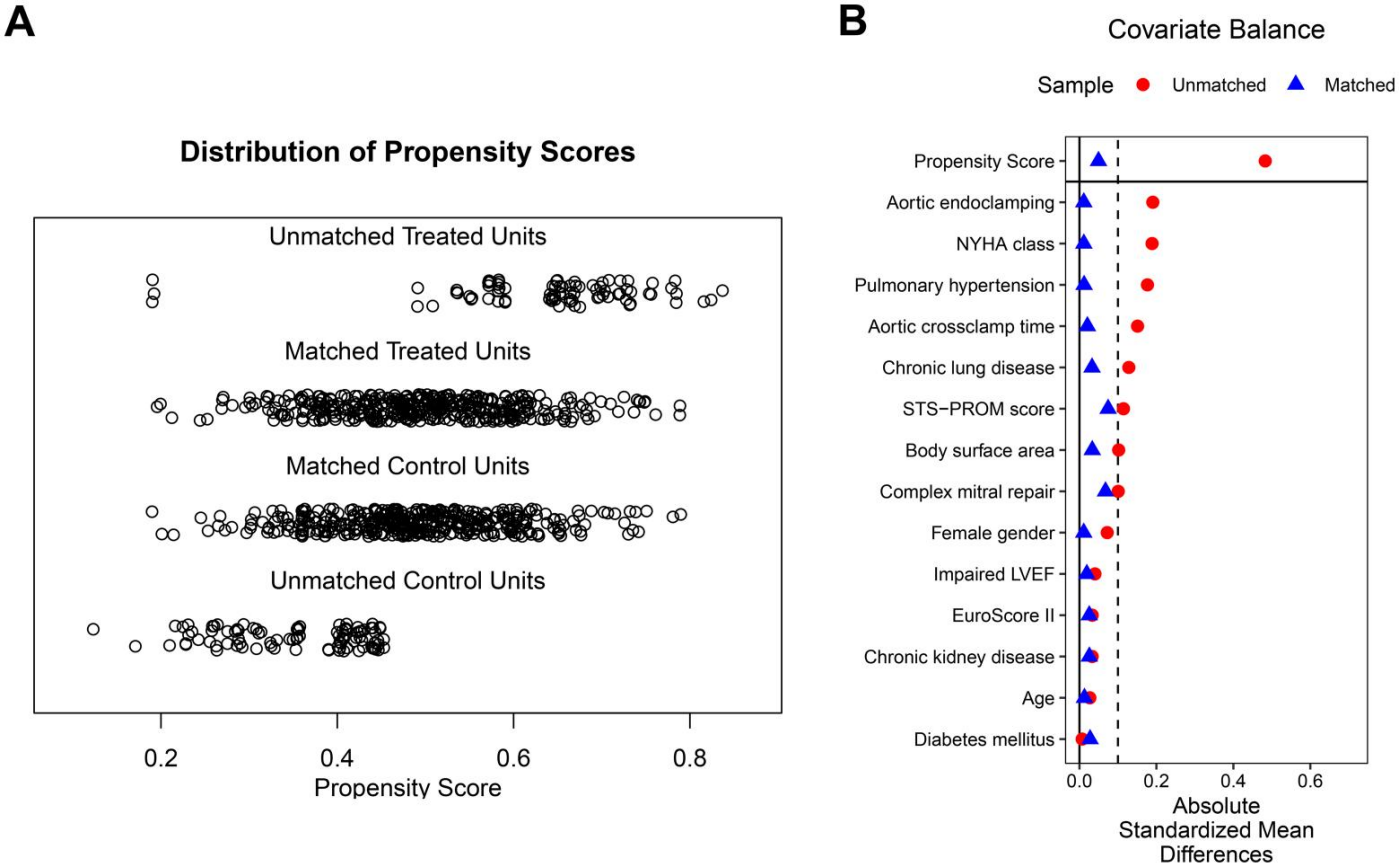
Distribution of Propensity Scores and Covariate Balance. (A) Jitter Plot of Estimated Propensity Scores before and after Matching; Unmatched Units Shown Separately. (B) Love Plot Showing Absolute Standardized Mean Differences (SMDs) before and after Matching. The Dashed Line Indicates the Predefined Balance Threshold (SMD = 0.1).

1:1 propensity score matching was performed using a generalized linear model (GLM) and nearest neighbour matching algorithm with a calliper width of 0.1 and without replacement, implemented via the *MatchIt* package in R.^20^ Matching variables included demographic and clinical risk factors (age, sex, body surface area, New York Heart Association functional class, left ventricular ejection fraction, chronic kidney disease, pulmonary hypertension, diabetes mellitus, EuroSCORE II, STS-PROM Score), as well as procedural parameters (aortic cross-clamp time, use of aortic endoclamping, and complexity of repair defined by leaflet pathology). No data were missing in preoperative covariates. Matching quality was assessed using standardized mean differences and Kolmogorov–Smirnov statistics (Love plot and Jitter plot in Figure 2).

Although blood samples were collected according to a standardized protocol, actual sampling times varied due to clinical workflow. For figures, serial CK and CK-MB measurements were smoothed using locally estimated scatterplot smoothing (LOESS) to depict group-level postoperative trajectories. To obtain interval-wise numeric summaries, we fitted a patient-specific natural cubic spline to CK and CK-MB values over time. For each patient and each prespecified window (0-12, 12-24, 24-48 hours), the spline was evaluated on a dense grid (0.1 hour) within the interval, and the time-averaged concentration was computed. For each interval and biomarker, patient-level time-averaged concentrations were compared between Del Nido and Custodiol using paired *t*-tests. We report group means ± SD (across patient summaries), the paired mean difference with its 95% CI, and *P*-values. Categorical data were expressed as absolute numbers and corresponding percentages. To control the family-wise error rate across the 6 primary comparisons (3 intervals × 2 biomarkers), Holm’s step-down adjustment was applied to *P*-values.

## RESULTS

### Study population

After exclusions, a total of 960 patients (Custodiol 485, Del Nido 475) were subjected to propensity score matching. After propensity score matching, 2 well-balanced cohorts of 389 patients each were generated, receiving either Custodiol or Del Nido cardioplegia. Groups were comparable in all baseline and procedural variables, except for cardioplegia type. A Love plot with detailed matching statistics, including standardized mean differences, is presented in Figure 2.

The mean age was 59 years, and preoperative risk profiles were similar between groups. The mean EuroSCORE II was 0.91% in the Custodiol group and 0.89% in the Del Nido group (*P* = .668), further confirming the cohort’s low-risk profile. Detailed baseline characteristics of the matching covariates are presented in Table 1.

**Table 1.** Baseline Characteristics of the Study Population

	Before PSM			After PSM		
Parameter	Custodiol *n* = 485	Del Nido *n* = 475	SMD	Custodiol *n* = 389	Del Nido *n* = 389	SMD
Age (years)	59.52 ± 13.15	59.86 ± 12.65	0.03	59.38 ± 13.00	59.22 ± 12.81	0.01
Female gender	133 (27.4%)	146 (30.7%)	0.07	117 (30.1%)	115 (29.6%)	0.01
Body surface area (m²)	1.95 ± 0.21	1.93 ± 0.22	0.10	1.95 ± 0.21	1.94 ± 0.22	0.03
EuroScore II (%)	0.92 ± 0.61	0.94 ± 0.72	0.03	0.91 ± 0.63	0.89 ± 0.56	0.03
STS-PROM Score (%)	0.63 ± 0.55	0.57 ± 0.51	0.11	0.60 ± 0.55	0.56 ± 0.51	0.07
Impaired LVEF	43 (8.9%)	37 (7.8%)	0.04	27 (6.9%)	29 (7.5%)	0.02
Pulmonary hypertension	79 (16.3%)	113 (23.8%)	0.18	73 (18.4%)	75 (19.4%)	0.01
Diabetes mellitus	18 (3.7%)	17 (3.6%)	0.01	14 (3.6%)	12 (3.1%)	0.03
Chronic lung disease	22 (4.5%)	12 (2.5%)	0.13	8 (2.1%)	10 (2.6%)	0.03
Chronic kidney disease	32 (6.6%)	28 (5.9%)	0.03	22 (5.7%)	27 (6.9%)	0.03
NYHA classification			0.19			0.01
Class 1	92 (19.0%)	103 (21.7%)		89 (22.9%)	74 (19%)	
Class 2	234 (48.2%)	258 (54.3%)		187 (48.1%)	218 (56.0%)	
Class 3	153 (31.5%)	114 (24.0%)		111 (28.5%)	97 (24.9%)	
Class 4	6 (1.2%)	0 (0.0%)		2 (0.5%)	0 (0.0%)	
Aortic endoclamping	100 (20.6%)	139 (29.3%)	0.19	95 (24.4%)	93 (23.9%)	0.01
Aortic cross-clamp time (min)	63.14 ± 20.78	60.08 ± 20.28	0.15	61.70 ± 19.22	61.28 ± 21.17	0.02
Complex mitral repair	82 (16.9%)	64 (13.5%)	0.1	62 (15.9%)	53 (13.6%)	0.07

Values are mean ± SD or *n* (%).

Abbreviations: LVEF, left ventricular ejection fraction; NYHA, New York Heart Association; PSM, propensity score matching; SMD, standardized mean difference.

### Procedural features

Procedural features were well balanced between the matched groups, with no relevant differences observed. Mitral valve repair typically involved ring annuloplasty with artificial chordae implantation, using consistent techniques throughout the study period. All patients underwent femoro-femoral cannulation for cardiopulmonary bypass. Percutaneous cannulation was more common in the Del Nido group, reflecting the institutional shift towards less invasive vascular access over time. Procedural data are summarized in Table 2.

**Table 2.** Procedural Characteristics

Parameter	Custodiol *n* = 389	Del Nido *n* = 389	*P*-value
Annuloplasty ring implanted	389 (100%)	389 (100%)	>.999
Leaflet repair (implantation of neochordae or partial resection) performed	332 (86%)	324 (83%)	.490
Percutaneous femoral cannulation	20 (5.1%)	137 (35%)	<.001
Aortic endoclamping	95 (24.4%)	93 (23.9%)	.926
Aortic cross‑clamp time (min)	61.70 ± 19.22	61.28 ± 21.17	.773
Cardiopulmonary bypass time (min)	95.00 ± 30.08	96.26 ± 34.15	.584
Procedure time (min)	146.42 ± 46.86	145.30 ± 45.46	.736
Cardioversion for ventricular arrhythmia after declamping	162 (41.6%)	55 (14.1%)	<.001
Number of shocks after declamping	0.8 ± 1.2	0.3 ± 1.3	<.001

Values are mean ± SD or *n* (%). *P*-values were calculated using paired *t*-tests for continuous and McNemar’s tests for categorical variables.

### Perioperative creatine kinase dynamics

Serial postoperative measurements of CK and CK-MB were used to assess myocardial protection. The analysis revealed consistently lower CK values in the Del Nido group compared to the Custodiol group during the first 48 postoperative hours. Within the first 12 hours, mean CK levels were markedly higher with Custodiol (766.0 ± 516.4 U/l) compared to Del Nido (614.0 ± 405.0 U/l, mean difference −152.1 [95% CI, −215.9 to −88.2], *P *< .001). This difference persisted across all predefined postoperative intervals.

**Figure 3.**
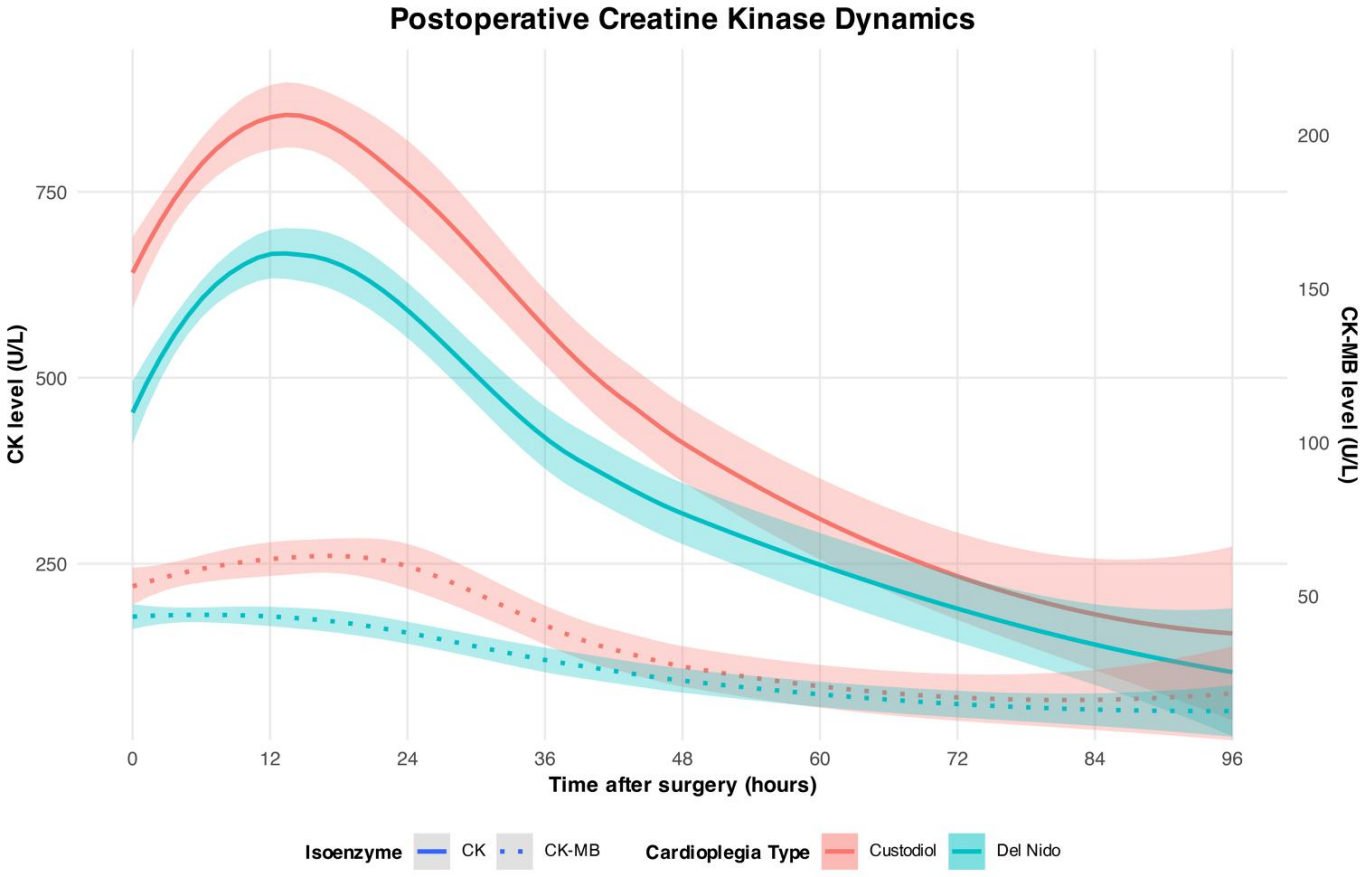
Postoperative Creatine Kinase Dynamics after Minimally Invasive Mitral Valve Surgery. LOESS-Smoothed Postoperative Serum Levels of CK (Solid Line) and CK-MB (Dotted Line) Comparing Custodiol (Red) and Del Nido (Blue) Cardioplegia. Lines Represent Mean Values with Shaded SE. Separate y-Axes Are Used Due to Differing Scales.

CK-MB levels showed a similar early pattern. Between 0 and 12 hours, Custodiol patients exhibited higher values (58.7 ± 63.1 U/l) compared with Del Nido (43.8 ± 32.1 U/l, difference −14.8 [95% CI, −21.9 to −7.7], *P *< .001). During 12-24 hours, CK-MB remained significantly higher with Custodiol (56.1 ± 96.3 vs 37.0 ± 43.5 U/l, difference −19.1 [95% CI, −29.7 to −8.5], *P *< .001). However, by 24-48 hours, the difference between groups was no longer significant (30.8 ± 141.9 vs 25.4 ± 45.1 U/l, *P *= .486). The complete interpolated biomarker trends are summarized in Table 3 and visualized in Figure 3.

**Table 3.** Creatine Kinase Dynamics in Custodiol vs Del Nido Cardioplegia

	CK (U/L)				CK-MB (U/L)			
postoperative time (hours)	Custodiol	Del Nido	Difference [95% CI]	*P*-value	Custodiol	Del Nido	Difference [95% CI]	*P*-value
0-12	766.0 ± 516.4	614.0 ± 405.0	−152.1 [−215.9 to −88.2]	<.001	58.7 ± 63.1	43.8 ± 32.1	−14.8 [−21.9 to −7.7]	<.001
12-24	766.6 ± 576.8	586.7 ± 450.8	−179.9 [−252.5 to −107.3]	<.001	56.1 ± 96.3	37.0 ± 43.5	−19.1 [−29.7 to −8.5]	<.001
24-48	560.2 ± 543.2	412.7 ± 532.6	−147.5 [−220.6 to −74.5]	<.001	30.8 ± 141.9	25.4 ± 45.1	−5.3 [−20.3 to 9.7]	.486

Values are patient-level time-averaged concentrations derived from natural cubic splines. Paired comparisons used dependent *t*-tests. *P*-values were Holm-adjusted across 6 comparisons (3 intervals × 2 markers); 95% CIs are unadjusted. Values are mean ± SD.

### Clinical outcomes

The perioperative minimum sodium concentration was higher (138.81 ± 8.06 vs 133.43 ± 3.74 mmol/l, *P* < .001), and cardioversion for ventricular tachycardia or fibrillation after declamping was required less frequently (14.1% vs 41.6%, *P* < .001) in the Del Nido group. When cardioversion was necessary, fewer shocks were delivered (0.3 ± 1.3 vs 0.8 ± 1.2, *P* < .001). Postoperative clinical outcomes are summarized in Table  4. Ventilation time, ICU stay, and hospital stay were similar between groups. In contrast, Del Nido was associated with more favourable perioperative parameters. The inotrope score at 6 hours was significantly lower (0.32 ± 1.71 vs 0.82 ± 2.25, *P* < .001). The vasoactive–inotropic score (VIS) at 24 hours did not differ between groups. Rates of acute kidney injury, perioperative pacemaker implantation, myocardial infarction, postoperative delirium, stroke, rethoracotomy for bleeding, and 30‑day mortality were low and comparable between groups (*P* > .28 for all). No deaths occurred within 30 days in either cohort (O/E ratio = 0.0 based on a mean EuroSCORE II of 0.9%).

**Table 4.** Clinical Outcome Measures

Parameter	Custodiol *n* = 389	Del Nido *n* = 389	*P*-value
Ventilation time (hours)	10.74 ± 20.06	11.35 ± 26.86	.585
Intensive care unit stay (hours)	39.25 ± 49.19	42.87 ± 67.28	.273
Primary hospital stay (days)	8.58 ± 4.71	8.78 ± 4.34	.516
Inotrope score (custom) 6 hours postoperative	0.82 ± 2.25	0.32 ± 1.71	<.001
Vasoactive inotropic score (VIS) 24 hours postoperative	1.20 ± 4.16	1.48 ± 11.21	.644
Acute kidney injury	2 (0.5%)	2 (0.5%)	>.999
Perioperative minimum sodium (mmol/L)	133.43 ± 3.74	138.81 ± 8.06	<.001
Postoperative delirium	11 (2.8%)	3 (0.8%)	.061
Perioperative pacemaker implantation	2 (0.5%)	6 (1.5%)	.289
Perioperative myocardial infarction	3 (0.8%)	3 (0.8%)	>.999
Perioperative stroke	5 (1.3%)	8 (2.1%)	.579
Rethoracotomy for bleeding	15 (3.9%)	18 (4.6%)	.710
30-day mortality	0 (0.0%)	0 (0.0%)	NA

Values are mean ± SD or *n* (%). *P*-values were calculated using paired *t*-tests for continuous variables and McNemar’s test for categorical variables.

## DISCUSSION

Our propensity-matched analysis demonstrates that Del Nido cardioplegia offers measurable advantages in myocardial protection over Custodiol in MIMVR. Patients receiving Del Nido had significantly lower postoperative CK and CK-MB release throughout the first two postoperative days, particularly within the first 24 hours, indicating reduced myocardial injury. Despite low absolute CK-MB values, the concordant reduction in CK and CK-MB suggests a biologically consistent effect. A higher rate of percutaneous cannulation in the Del Nido group may have contributed to slightly lower total CK values, as this technique is associated with less local surgical trauma. An exploratory analysis did not demonstrate a significant independent effect of percutaneous cannulation on postoperative enzyme release. Apart from cardioplegia strategy and cannulation technique, no relevant changes in surgical technique, perioperative management, or postoperative care occurred during the study period that would be expected to systematically influence CK release. The observed CK differences are therefore most plausibly attributable to cardioplegia strategy. Additionally, the Del Nido group required less inotropic support at 6 hours, maintained higher perioperative serum sodium levels, and experienced a markedly lower incidence of ventricular fibrillation upon reperfusion (14.1% vs 41.6%, *P *< .001). Although no formal changes in perioperative or intensive care protocols occurred during the study period, temporal changes in inotrope use cannot be excluded and should be considered when interpreting these results. Importantly, these benefits did not affect major clinical endpoints or 30-day mortality, which were low and similar between groups. In summary, Del Nido cardioplegia demonstrated superior biomarker dynamics and fewer ventricular arrhythmias post-cross-clamp, while major postoperative outcomes showed no significant differences.

These results align with and expand upon the emerging literature comparing single-dose cardioplegia in MIMVS. Kang et al.^12^ reported in a smaller matched cohort (*n* = 312) that Custodiol was associated with higher postoperative CK/CK-MB release on postoperative day 2, although effects were less pronounced than in our study. Our larger cohort confirms comparable clinical outcomes with a more pronounced enzymatic advantage. Similarly, Lee et al.^21^ found lower peak CK-MB and troponin I with Del Nido compared with HTK in minimally invasive cases, with equivalent early outcomes. Their regression suggested benefit up to ∼100 min ischaemia, though data on redosing Del Nido remain inconsistent. Malvindi et al.^22^ (*n* = 120) found similar enzymes and outcomes, but several practical advantages for Del Nido: one-third cardioplegia volume, less ultrafiltration, and more spontaneous sinus rhythm after unclamping. A 2022 network meta-analysis by Tan et al.^13^ found Del Nido associated with the lowest perioperative mortality among 4 strategies. Taken together, the present findings, when considered alongside the existing literature, suggest that Del Nido cardioplegia is at least equivalent to Custodiol with respect to early myocardial preservation, with growing evidence of favourable biomarker and electrophysiological profiles in minimally invasive surgery.

Custodiol provides prolonged arrest with a single dose but requires large volumes (1.5-2 L) that may promote dilutional hyponatraemia. In our series, sodium nadir was lower with Custodiol. By contrast, Del Nido uses near-normal sodium plus magnesium and lidocaine, causing less haemodilution and more stable electrolytes.^22^ Postoperative delirium was less frequent with Del Nido in our cohort, although statistical significance was not reached. The more favourable enzyme kinetics observed with Del Nido may be partly explained by the presence of blood in the solution, which provides residual oxygen to support transient aerobic metabolism during the ischaemic period. While both solutions can be used in a single-dose fashion in MIMVS, Del Nido may require redosing beyond 90 min. Yücel et al.^23^ showed worse myocardial injury markers when Del Nido exceeded 90 min without redosing, highlighting uncertainty about redosing protocols in longer procedures. Importantly, redosing strategies are heterogeneous and largely dependent on individual surgeon discretion, reflecting the lack of robust evidence guiding optimal redosing protocols. In routine MIMVS, however, clamp times are well covered by a single Del Nido dose. Furthermore, procedures requiring repeated cardioplegia dosing or repeated cross-clamping are typically more complex and have been associated with less favourable outcomes, independent of cardioplegia choice. To ensure standardized conditions and minimize confounding, such cases were therefore excluded from the present analysis. Another advantage of Del Nido cardioplegia is its ability to be prepared in-house, whereas Custodiol is a commercial product, making Del Nido the more cost-efficient option. A further distinction lies in arrhythmia incidence. Custodiol’s hyperpolarizing arrest is linked to reperfusion ventricular fibrillation.^9^ In our study, >40% required cardioversion, vs ∼14% with Del Nido. This marked reduction in reperfusion arrhythmias suggests greater myocardial electrical stability and may contribute to lower enzyme release.

It must be emphasized that the present analysis reflects outcomes under predefined and standardized intraoperative conditions. In clinical practice, procedural complexity, cross-clamp duration, or the need for repeated cardioplegia dosing may only become apparent during surgery. The current study was not designed to evaluate such scenarios and therefore cannot determine whether Del Nido performs differently from Custodiol in unselected cases. Accordingly, our findings should not be interpreted as a universal recommendation across all levels of procedural complexity. Future studies focusing on prolonged ischaemia and redosing strategies are required. Overall, in this retrospective analysis, Del Nido cardioplegia demonstrated superior myocardial protection compared to Custodiol in low-risk MIMVR for type II pathology and may be considered the preferred cardioplegic solution in this rapidly evolving surgical setting.

## LIMITATIONS

This retrospective, single-centre study is subject to selection bias, and unmeasured confounders cannot be excluded. However, strict propensity score matching on key procedural and baseline variables was performed to minimize these effects. As Del Nido was introduced later, time-related bias, including gradual improvements in surgical experience, team proficiency, and perioperative management, cannot be entirely excluded in a single-centre setting, although no major changes occurred in institutional protocols or perioperative management during the study period. Most surgeons had already surpassed their individual learning curve. Residual temporal effects may persist despite matching and should be considered when interpreting the results. Cases with multiple cardioplegia doses or repeated cross-clamping were excluded, as redosing schemes were heterogeneous, largely dependent on individual surgeon discretion, and typically associated with complex procedures. The analysis therefore focused on standardized single-dose conditions. Postoperative myocardial injury was assessed using CK and CK-MB, which represent well-established biomarkers for longitudinal postoperative monitoring in cardiac surgery. Cardiac troponin was not routinely available for analysis. The primary outcome measures represent surrogate markers of myocardial injury and intraoperative course and should not be interpreted as direct indicators of long-term clinical benefit. The cohort was deliberately limited to low-risk, elective, isolated mitral repairs, restricting generalizability to higher-risk or complex cases. These findings require confirmation in a larger, ideally prospective randomized trial.

## CONCLUSIONS

In MIMVR, both Custodiol and Del Nido provided excellent clinical outcomes. In this propensity-matched cohort of low-risk, mitral valve repairs, Del Nido showed lower release of postoperative surrogate markers of myocardial injury and fewer reperfusion ventricular arrhythmias with no increase in adverse clinical outcomes and no inference regarding long-term clinical benefit. For routine MIMVR cases, Del Nido may be considered the preferred cardioplegic solution. If a prolonged cardioplegic arrest is anticipated, it remains uncertain whether to redose Del Nido or prefer Custodiol. Prospective randomized trials are needed, particularly in prolonged ischaemia and higher-risk patients.

## Data Availability

The data underlying this article cannot be shared publicly due to data protection issues. The data will be shared on reasonable request to the corresponding author.
